# Fluid Containing Structures in the tips of the fingers and toes delineated by Ultrasound Imaging before and after Induced Skin Wrinkling

**DOI:** 10.1038/s41598-018-38476-5

**Published:** 2019-02-07

**Authors:** Antonin Gechev

**Affiliations:** Royal Free London Hospital and West Hertfordshire Hospital, Department of Clinical Neurophysiology, London, Pond Street NW3 2QG UK

## Abstract

This Ultrasound study identified spaces within the pulp of distal phalanx of the fingers and toes that halve in area after Water Induced Skin Wrinkling. These spaces appeared as fluid filled sacculi between connective tissue compartments accountable for distending digital pulp under normal circumstances and skin wrinkling after water immersion. Whilst studying conditions related to sympathetic nerve function with WISW ultrasound imaging is a valuable adjunct to the visual assessment.

## Introduction

The wrinkling of fingers and toes after immersion in water or after topical application of EMLA, has been studied for over seven decades^1^, and it can be diagnostically useful. A reduction in wrinkling has been observed in peripheral nerve dysfunction^2^, and an increase observed in cystic fibrosis^3^. Current clinical practice recognizes a 4-grade visual scale of wrinkling^4^, but an alternative quantification of the changes by using ultrasound imaging might provide an improved grading of the changes.

The aim of this study was to explore structural digital pulp changes in the fingertips and tips of the toes by Ultrasound and Power Doppler Imaging before and after water induced skin wrinkling (WISW).

## Results

### Visual assessment

**Figure 1:**
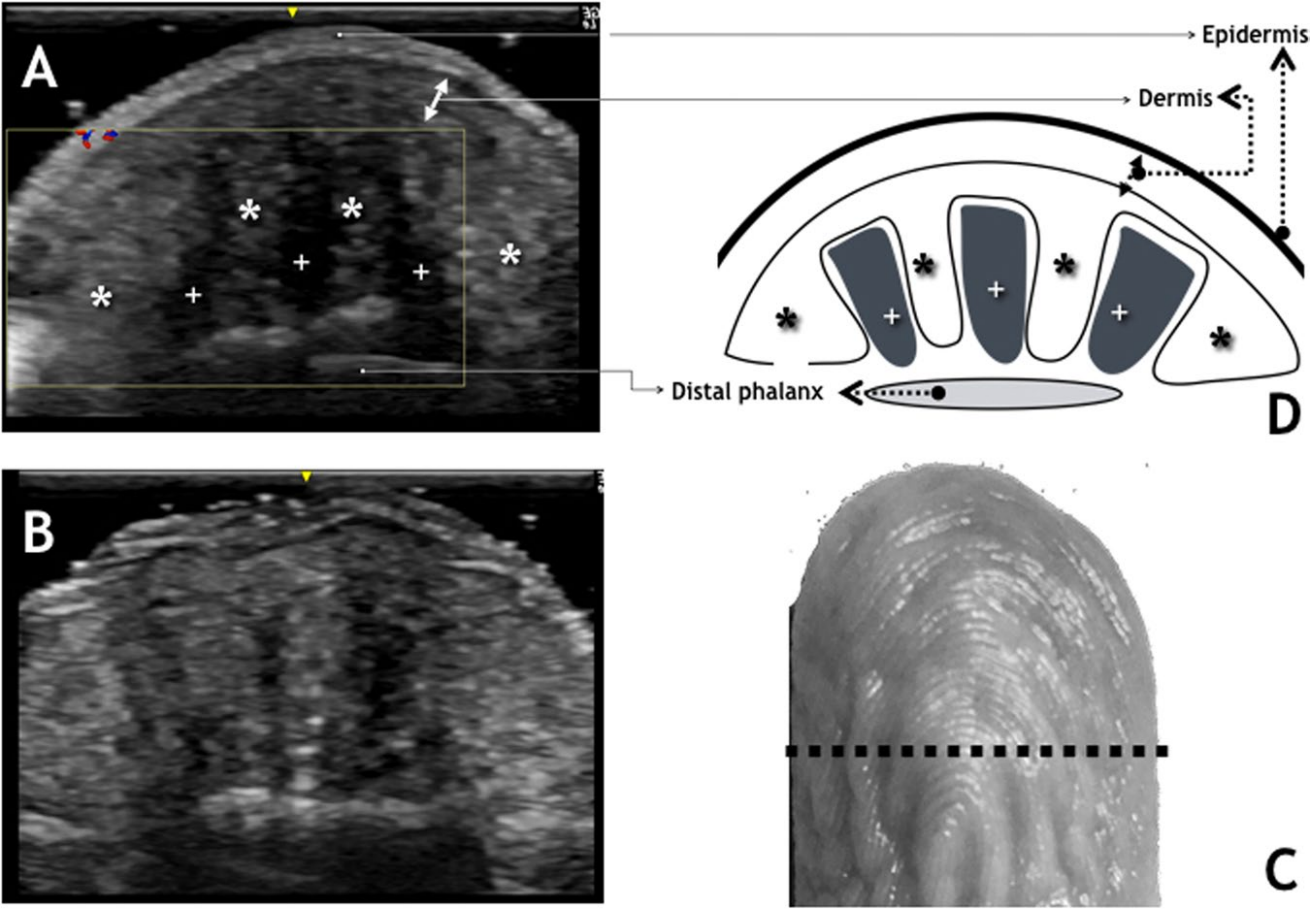
Middle Finger – transverse view. (**A**) US image Before WISW. (**B**) US image After WISW. (**C**) Photograph of the middle finger after WISW with dotted line representing the scanning plane and length. (**D**) Drawing depicting the structures described in the text. ^*^Hyper-echogenic areas with pillar like structures. ^+^Hypo-echogenic areas.

**Figure 2:**
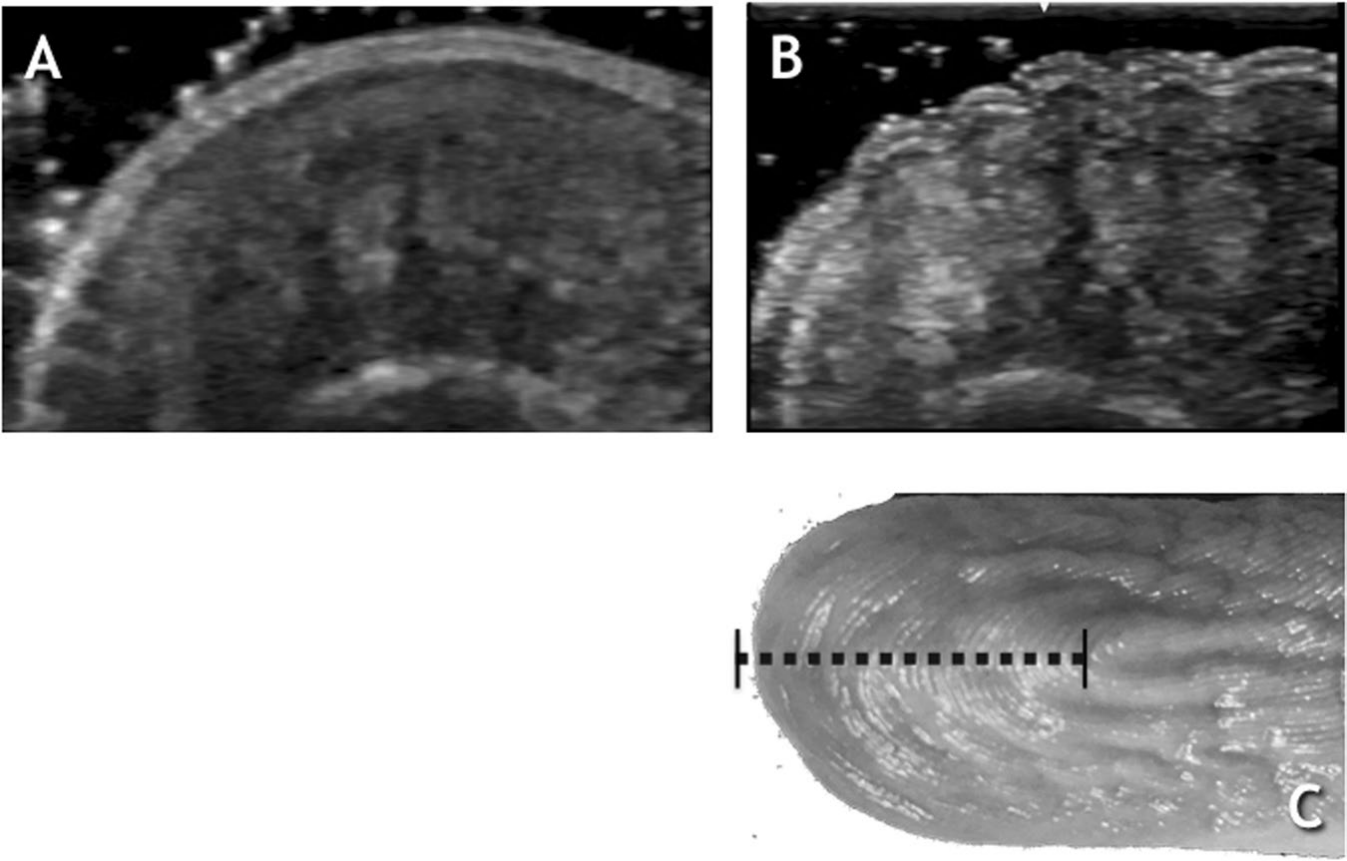
Longitudinal view of the Middle Finger shown in Fig. 1. (**A**) US image Before WISW. (**B**) US image After WISW. (**C**) Photograph of the middle finger after WISW with dotted line representing the scanning plane and length.

**Figure 3:**
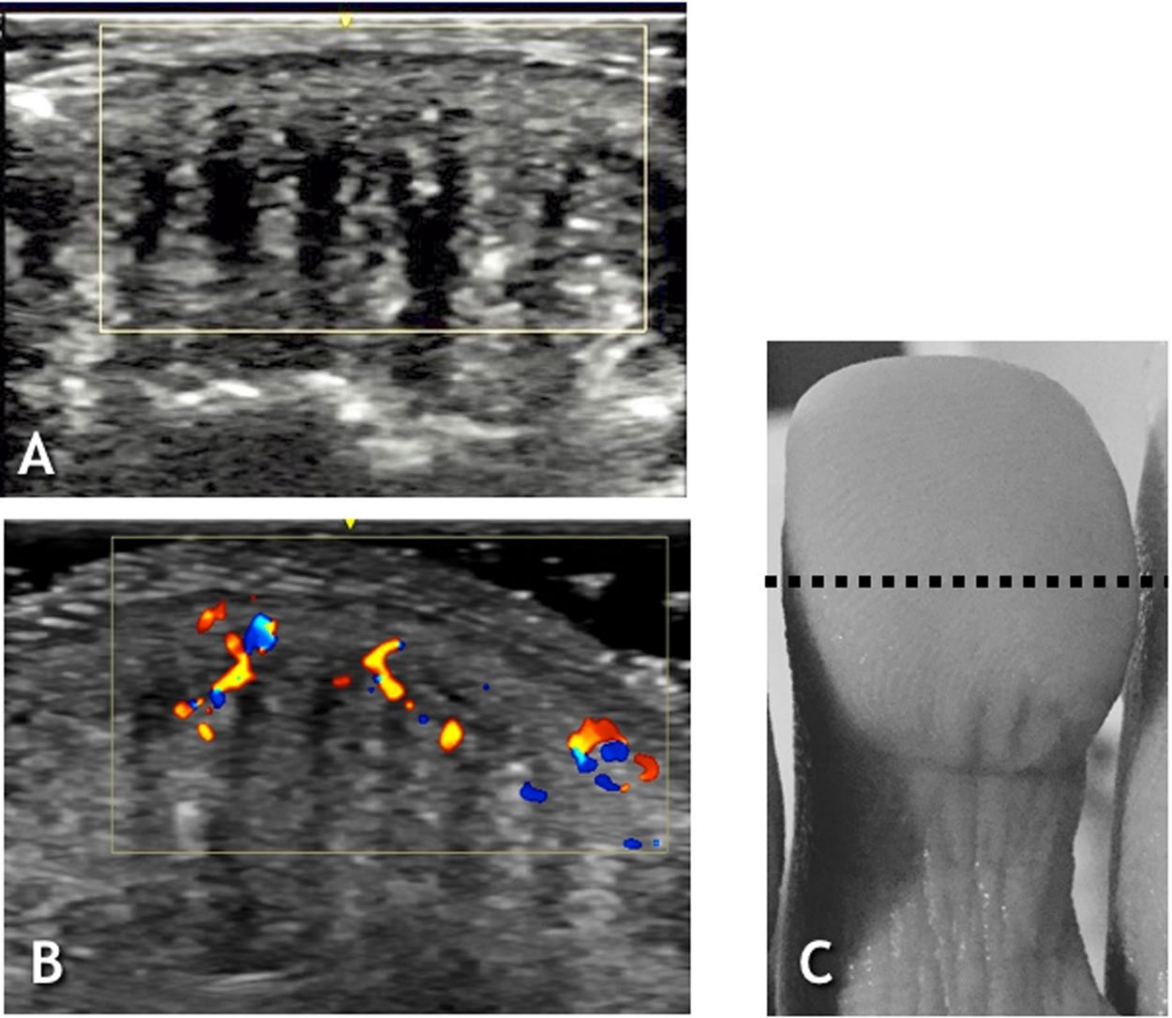
Second Toe – transverse view. (**A**) US image Before WISW. (**B**) US image After WISW. (**C**) Photograph of the middle finger after WISW with dotted line representing the scanning plane and length.

**Figure 4:**
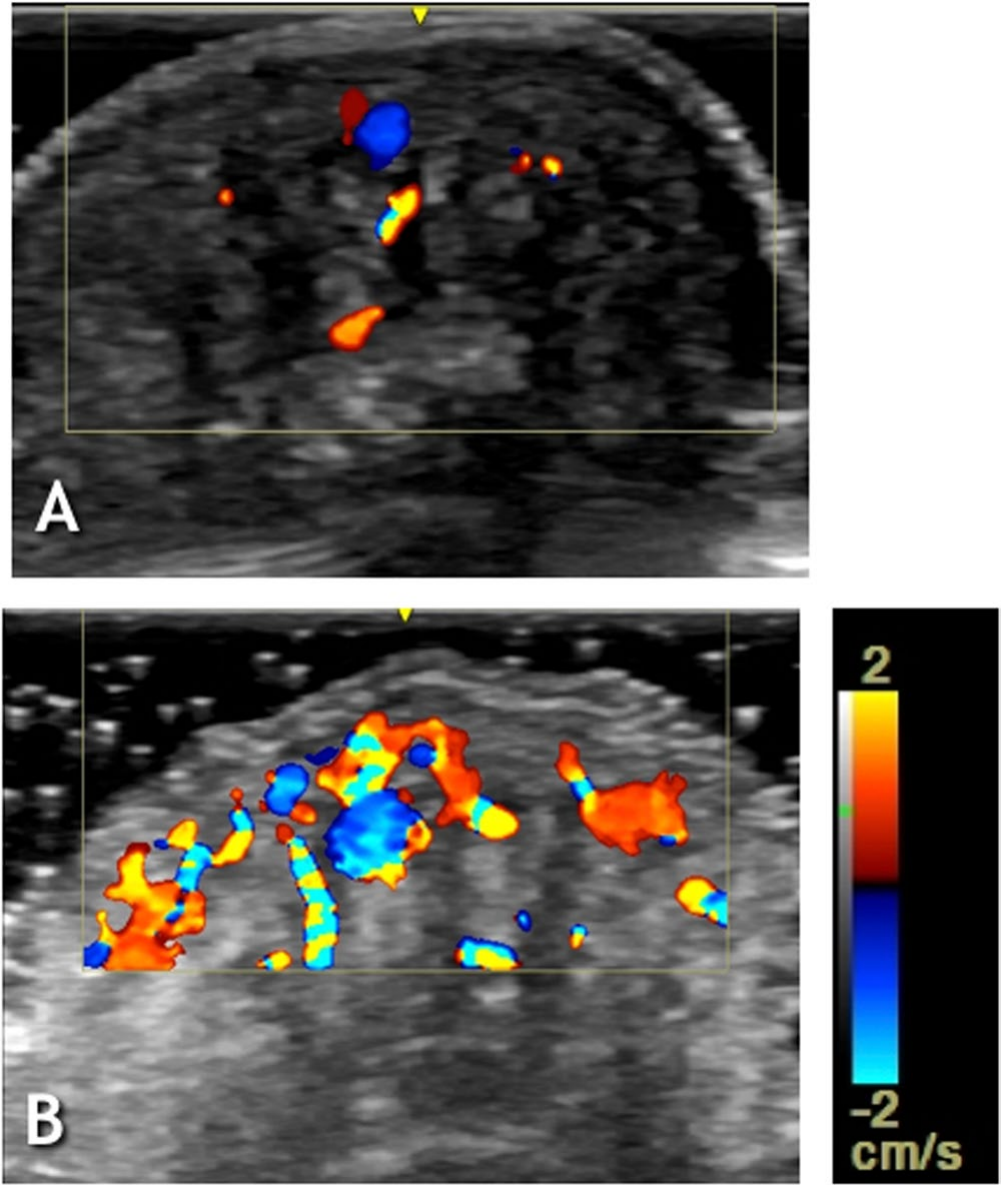
Index finger – transverse view. (**A**) US and Power Doppler Imaging Before WISW. (**B**) US and Power Doppler Imaging After WISW. Please note increased color flow signal after WISW in the dermis and hyper-echogenic areas.

The ultrasound imaging at the end of the distal finger (toe) phalanx (DF(T)P), showed 4 to 8 subcutaneous (attached to each other towards the dermis), hyper-echogenic pillar like structures with triangular or rectangular shape, perpendicularly orientated to the convex, volar skin surface, “arching” the hypo-echogenic spaces (Fig. 1D). This pattern was similar in both transverse and longitudinal scanning planes (Figs 1A, 2A, 3A). Color and Power Doppler Imaging (PDI) showed color-flow signal mainly in the hyper-echogenic regions (Fig. 4).

### Quantitative assessment

#### Prior to water immersion

The average cross sectional area of the subcutaneous hyper-echogenic regions before Water Induced Skin Wrinkling (WISW) was 48.4% of the total distal finger (toe) phalanx (DF(T)P) soft tissue cross sectional area as measured on the transverse scanning plane US images. The average hypo-echogenic space cross sectional area was 12.9% of the total distal finger (toe) phalanx soft tissue cross sectional area. Power Doppler scaling showed Grade 0–1 color flow signal in the same stored images used for visual assessment.

#### After water immersion

The total cross sectional soft tissue DF(T)P area did not change significantly after WISW nor did the cross sectional area of subcutaneous hyper-echogenic regions (Table 1). In contrast, the mean hypo-echogenic area showed significant decrement after WISW. Thus, the average hypo-echogenic space reduced from 12.9% to 6.6% of the total distal finger (toe) phalanx soft tissue cross sectional area.

**Table 1:** Mean values and SD of wrinkles and Ultrasound DF(T)P US measurements Pre- and Post-WISW.

	Hyper-echogenic pillar-like structures	Wrinkles fingers	Hyper-echogenic structures area (cm^2^)		Hypo-echogenic space area (cm^2^)		Total FD(T)P area (cm^2^)	
			Pre	Post	Pre	Post	Pre	Post
DF(T)P	n = 39	n = 21	n = 27	n = 27	n = 27	n = 27	n = 27	n = 27
Mean	5.79	5.1	0.30	0.31	0.08	0.04	0.62	0.61
SD	0.97	0.90	0.05	0.05	0.02	0.01	0.06	0.06
P value			p = 0.13*		**p < 0.001** ^**#**^		p = 0.67*	

DF(T)P – Distal Finger (Toe) Phalanx.

n = number of fingers and toes.

*Paired Samples t-Test.

^#^Wilcoxon Signed Ranks Test.

The individual hyper-echogenic pillar-like structures appeared attached to each other (with less distinguishable boundaries) as the hypo-echogenic spaces between them flattened or faded, resulting in slight, but not significant increase in the hyper-echogenic area measurement. The pre-wrinkling mean hyper-echogenic pillar-like structures number was the same as the mean wrinkles number.

To summarise, the following structural changes can be described:

1. A dorso-ventral retraction with convergence to the midline of the hyper-echogenic pillar like-structures, whilst hypo-echogenic spaces between them flattened, reflected in epidermal depressions (wrinkles);
2. Near twofold reduction in hypo-echogenic spaces (Figs 1B, 2B, 3B); Table 1;
3. Increased color-flow signal within the hyper-echogenic regions from Grade 0–1 to Grade 2 color-flow signal as indicated by Power Doppler Imaging.

## Discussion

This Ultrasound study has identified spaces within the pulp of the distal phalanx of the fingers and toes that approximately halve in area after WISW. These spaces appear as homogenous, hypo-echogenic areas separating the adjacent heterogeneous, hyper-echogenic areas in both sagittal and coronal planes.

Uniform Ultrasound hypo-echogenicity can occur with adipose tissue or with fluid, whilst varied hyper-echogenicity usually reflects mixed, connective tissue^5^. Because fat exists in solid, liquid and melting phases depending on the temperature, with a transitional phase occurring at 35 °C^6^, the immersion with warm water could reflect a change of the state of fat from solid to liquid; however in that event no volume loss is to be expected with a rise in temperature. Thus, the observed shrinkage of the hypo-echogenic spaces after WISW implies their content is fluid rather than a solid or stiff substance. Anatomically, subcutaneous tissue is lobulated in compartments in the most distal sections of the fingers^7^. The hypoechogenic areas appear on the US images as elongated triangles or rectangles, most likely because they occupy the spaces parallel to the fibrous septi which separate connective tissue compartments in a ventro-dorsal direction, connecting the dermis to the periosteum^8^. Thus, in a two dimensional image the “walls” of hypoechogenic spaces would take the shape of the “inner” surface of the connective tissue compartments, insulated by the fibrose speti. Hence, it is plausible that these “spaces” are organised as fluid filled sacculi distending digital pulp under normal circumstances, thereby smoothing the skin. The Power Doppler Imaging did not show changes attributable to haemodynamic factors either arterial or venous that might confound the above mentioned conclusions. An earlier Ultrasound 8 MHz study^9^ has not described such a morphological feature in the digital pulp, neither have Computed Tomography; Magnetic Resonance Imaging nor cadaver fingers studies^7^.

The hypoechogenic areas occupy the space below the dermis extended to the distal phalanx bone so they are unlikely to represent a structural array of glomus bodies which are buried from 0.5 to 1 mm below the deepest layer of the epidermis^10^. Furthermore, no blood flow signal was detected by PDI in the hypoechogenic spaces either pre- or post- WISR to suggest they are composed of blood vessels. But it is difficult to determine the nature of the fluid substance based solely on the US images. The distal phalanx angiograms and phlebograms studies do not visualise vascular structures other than typical anastomotic arcades at the fingertips^8^. These arterial and venular arcades do not resemble hypoecogenic areas seen in the current US study. Furthermore, PDI did not show blood-flow signal within the hypoecogenic areas so it could be hypothesized that their fluid content is extravascular, i.e. serum or lymph.

The retraction/convergence in DF(T)P of connective tissue compartments after WISW was associated with significant waning of space between them, and this accords with the hypothesis of digital pulp volume loss as the main determinant of induced skin wrinkling^2^. However, it is important to distinguish whether pulp volume loss is intra- or extravascular or a combination of both. The US data presented here depict a fluctuating volume contained in fluid filled structures in the pulp, with no evidence from PDI of microvascular changes within them, which is in favour of extravascular volume loss after WISW. The previously proposed reduction in tissue/vessel mass due to differential vasoconstriction^11^ has a different impact - loss of intravascular volume as well as drop in digital pulp pressure. It appears that both extravascular and intravascular mechanisms of volume loss are involved in WISW.

Further studies are needed to clarify how extravascular fluid is formed and enters the hypoechogenic areas as well as whether these spaces are somehow related to the recently discovered “Unrecognised Interstitium” in human tissues^12^. The distinctive glomus bodies might be also involved in those processes, beside their known mechanoreceptor and thermoregulatory functions^13^. On the other hand, the conservation of pilar like structures area whilst hypoechogenic areas constrict after WISW suggests the extravascular fluid is “drained off” the digital pulp rather than “buffered” into the neighbouring connective tissue compartments. The physiological way to siphon off this fluid would be solely through the deep dermal lymphatic plexuses, the vessels of which are considerably wider-bored with typical moniliform appearance characteristic of the presence of valves^10^. The wrinkling in adults initiates at 4 to 10 min after immersion (depending on the water temperature)^14^, so the speed of wrinkling might be also linked to the velocity of lymph flow (ranging from 100 to 1500 um/sec in animals)^15^.

The Ultrasound measurements provide an alternative means of quantifying skin wrinkling at the level of fingertip. The count should correspond to the number of hyper-echogenic regions each surrounded by one hypo-echogenic area. When no visible wrinkling is seen after water immersion, as in the toes, the reduction in area of the hypoechogenic structures visualized on ultrasound provides an indirect estimate of fluid loss in the distal phalanx. The effectiveness of ultrasound in assessing WISW in the toes overcomes the current clinical limitation of reliable visual assessment being confined to the hands. The feasibility of including the toes exposes long nerve fibres to measurements potentially pertinent to the study of length dependent small fibre peripheral neuropathies.

## Methods

11 hospital staff volunteers aged 22–53 (8 female and 3 male) with no history of neuropathy, carpal or tarsal tunnel syndrome were included in this study. All participants gave their informed consent. The study was approved by Health Research Authorities (IRAS 221584) and all research was performed in accordance with relevant guidelines/regulations.

The ultrasonic examinations were carried out by longitudinal and transverse High Frequency (22 MHz) Ultrasound and Power Doppler Imaging (PDI) using LOGIQ e Ultrasound GE machine. The US device settings - gain, compression, focus, time gain compression as well as US probe position were kept constant throughout the assessments. The Ultrasound and Power Doppler Imaging was conducted before and after water immersion as follows:

Hands: Subjects were seated in front of the examiner with the dorsal hand surface stabilised on a pillow. The ultrasound probe was held perpendicular to the volar skin surface at the level of distal phalanx of index, middle and ring fingers (11 subjects).

Feet: The subjects were lying prone on a bed with the dorsal foot surface resting on a pillow. The ultrasound probe was held perpendicular to the plantar skin surface at the level of the distal phalanx of second and third toe (3 subjects).

The ultrasound probe surface was kept a few millimetres above the skin level without pressure on to the finger or toe surfaces. This was controlled by visual feedback from the US screen leaving a contact gel filled gap between the probe and the skin. Thus, phalanx convexity was not altered during scanning.

The subjects were asked to immerse their hand and/or foot in a bowl of warm water −38 °C for 15–30 mins. Skin wrinkling was assessed visually and the number of wrinkles per finger was noted. No numbers were recorded for skin wrinkling of the toes because of practical difficulties in arriving at a reliable count. Seven of the eleven subjects participated in WISW.

### US Imaging measurements

The Ultrasound and Power Doppler images were taken from the volar/plantar phalanx surface, distal to the eponychium, in both transverse and longitudinal planes:

(A) Transverse – perpendicular to the lateral nail folds; (B) Longitudinal - parallel to the lateral nail folds, at the midline. Imaging started with cross sectional view at the tuberosity of the third phalanx searching for the end of distal phalanx bone echo as internal landmark. At this level the US transducer was slightly angulated as focal areas of interest became apparent. Then images were stored for offline qualitative and quantitative analysis. The pulp tissue layers were distinguished visually according to their echogenicity and outlined manually with the incorporated software cursor. Then cross-sectional area software function of the ultrasound device was used to quantify outlined layers area in cm^2^. The area of each individual layer was expressed as a percentage of the total pulp area.

The Power Doppler Imaging scores adopted were: Grade 0: no color-flow signal; Grade 1: Spotty color-flow signal; Grade 2: Flare-like color-flow signal; Grade 3: Reticular color-flow signal^16^.

Statistical analysis. SPSS (v24) software package was used for the calculations of the mean and standard deviations; difference of means before and after WISW was tested using Paired Samples T-test as well as Wilcoxon Signed Ranks Test.

## Data Availability

The datasets generated during and/or analysed during the current study are available from the corresponding author on reasonable request.
